# Preventive efficacy of oral moxidectin at various doses and dosage regimens against macrocyclic lactone-resistant heartworm (*Dirofilaria immitis*) strains in dogs

**DOI:** 10.1186/s13071-019-3685-3

**Published:** 2019-09-11

**Authors:** Tom L. McTier, Robert H. Six, Aleah Pullins, Sara Chapin, Kristina Kryda, Sean P. Mahabir, Debra J. Woods, Steven J. Maeder

**Affiliations:** Zoetis, Veterinary Medicine Research and Development, 333 Portage St, Kalamazoo, MI 49007 USA

**Keywords:** *Dirofilaria immitis*, Heartworm, Canine, Macrocyclic lactone, Resistance, Moxidectin

## Abstract

**Background:**

Moxidectin has previously shown limited efficacy (≤ 44.4%) against confirmed macrocyclic lactone (ML)-resistant *Dirofilaria immitis* strains at 3 µg/kg after single and multiple oral dosages. Three studies were conducted to evaluate higher oral moxidectin doses for efficacy against confirmed ML-resistant *D. immitis* strains.

**Methods:**

Dogs were inoculated with 50 *D. immitis* L3 and randomly allocated to treatments. Study 1: 6 groups of dogs (*n* = 8) were inoculated with JYD-34 (Day − 30) and treated as follows: T01, negative control; T02–T05, moxidectin at 3, 6, 12 or 24 µg/kg, respectively, on Day 0 only; T06, moxidectin at 3 µg/kg on Days 0, 30 and 60. Study 2: 10 groups of dogs (*n* = 5) were inoculated (Day − 30) with either JYD-34 (T01, T03–05) or ZoeLA (T02, T06–T10) and treated as follows: T01 and T02, negative controls; T03–T05, moxidectin at 24, 40 or 60 µg/kg, respectively, on Days 0, 28 and 56; T06 and T09, moxidectin at 3 or 60 µg/kg on Day 0 only; T07, T08 and T10, moxidectin at 24, 40 or 60 µg/kg, respectively, on Days 0, 28 and 56. Study 3: 5 groups of dogs (*n* = 5) were inoculated with ZoeMO (Day − 28) and treated as follows: T01, negative control; T02, moxidectin at 3 µg/kg moxidectin on Day 0 only; T03–T05, moxidectin at 24, 40 or 60 µg/kg, respectively, on Days 0, 28 and 56. All dogs were necropsied for adult heartworm recovery ~ 4–5 months post-inoculation.

**Results:**

All moxidectin-treated dogs showed significantly lower worm counts than controls. The efficacy of moxidectin administered once at 3 µg/kg was 19% (JYD-34), 44.4% (ZoeLA) and 82.1% (ZoeMO). Increasing both the dose and the number of dosages of moxidectin improved efficacy, with 100% protection obtained using three dosages of moxidectin at either 40 µg/kg (JYD-34, ZoeMO) or 60 µg/kg (ZoeLA). Three dosages of 24 µg/kg were also highly effective, providing ≥ 98.8% efficacy for all three strains.

**Conclusions:**

Increasing both the dose and number of consecutive monthly dosages of moxidectin improved the efficacy against ML-resistant heartworms. Based on these data and other technical considerations, the 24 µg/kg dose was considered the optimal dose for further commercial development.

## Background

Macrocyclic lactones (MLs) have been used to effectively and safely protect dogs against heartworm (HW) disease for more than 30 years [1–11], with ivermectin (Heartgard^®^, Merial, Duluth, GA, USA), selamectin (Revolution^®^/Stronghold^®^, Zoetis, Parsippany, NJ, USA), moxidectin (ProHeart^®^ 6/SR-12, Zoetis, Parsippany; Advantage Multi^®^, Bayer Animal Health, Shawnee, KS, USA) and milbemycin oxime (Interceptor^®^, Elanco, Greenfield, IN, USA) historically providing 100% efficacy against *Dirofilaria immitis* when used as recommended. However, canine HW disease incidence continues to increase in many countries around the world including the USA [12–16]. The Companion Animal Parasite Council reported a 15.3% increase in cases of dogs positive for *D. immitis* between 2013 and 2016 [17], and the American Heartworm Society (AHS) reported an increase of 21.7% in the average number of cases per clinic over the same time period [16, 18]. This rise in incidence is concerning but not necessarily surprising given that it is likely that less than 35% of the 70 million pet dogs in the USA are regularly tested for *D. immitis* infection and up to 70% do not receive a regular HW preventive [13].

Reports of ML lack of efficacy (LOE) in the field have increased in the last ten years [19–24] and the lack of regular and correct administration of available preventives is a major contributor to this problem [13, 16, 19, 24]. In their analysis of 271 LOE reports from the USA involving dogs on HW prevention, Atkins et al. [19] found insufficient preventive was purchased by owners to provide AHS-recommended year-round protection in more than 80% of the cases. The emergence of *D. immitis* populations resistant to ML treatment has added a new dimension to this already complex problem. At present, efficacy data collected in both the laboratory and field confirm ML resistance is a growing problem [25–33], and analyses conducted on various *D. immitis* isolates/strains have confirmed genetic differences that are associated with the resistance phenotype [20, 27, 34–37]. The confirmation of ML resistance in these strains, in conjunction with the possibility of as yet unidentified strains circulating in the field, raises concerns for the future viability of present canine HW disease prevention methods and underscores the need to define the extent of ML resistance in *D. immitis* and develop new approaches to prevention.

Moxidectin is a ML presently available in HW preventive products worldwide that has a proven track record of preventing HW disease [2, 3, 5, 7, 8, 38]. Within the USA, moxidectin is marketed in two presentations, topical (Advantage Multi^®^ and Coraxis™, Bayer Animal Health) and injectable (ProHeart^®^ 6 and ProHeart^®^ 12, Zoetis, Parsippany). Moxidectin was also previously approved as an oral formulation and is still marketed as such outside the USA (e.g. ProHeart^®^ tablets, Zoetis, Parsippany). All four formulations provided 100% protection against ML-susceptible *D. immitis* strains with a single dose on original approval and in subsequent laboratory studies [1–3, 5, 7, 8, 32, 38]. Moxidectin has also been shown to have activity against a number of ML-resistant *D. immitis* strains in all three formulations and it often performs equally well or better than other MLs against ML-resistant strains [39]. For example, the JYD-34 *D. immitis* strain has been confirmed genetically and in laboratory efficacy studies to be highly ML-resistant, but one administration of Advantage Multi^®^ provided dogs with 100% protection against the strain [25] and a single ProHeart^®^ 6 injection provided 99.5% efficacy [29]. Additionally, three monthly dosages of oral moxidectin at the minimum recommended label dose (3 µg/kg) were 44.4% effective against JYD-34 [32], which is higher than the efficacy obtained with three dosages of either ivermectin (6 µg/kg) (29.0%, as Heartgard^®^ Plus) or selamectin (6 mg/kg) (28.8%, as Revolution^®^) and similar to the protection provided by three dosages of milbemycin oxime (0.5 mg/kg) (52.2%, as Trifexis^®^, Elanco, Greenfield, IN, USA) [25]. In addition to JYD-34, a single dose of oral moxidectin (3 µg/kg) has shown efficacy against the resistant ZoeLA, AMAL and ZoeMO *D. immitis* strains, providing dogs with 54.0%, 61.6% and 82.7% preventive efficacy, respectively [32]. Recent work has also highlighted the microfilaricidal activity of moxidectin against the ZoeMO *D. immitis* strain, with more than 95% reduction in circulating microfilariae reported for infected dogs within 12 weeks of treatment with ProHeart^®^ 6 or ProHeart^®^ 12 compared to control dogs [31]. Finally, recent data from a large field study of client-owned dogs demonstrated that ProHeart^®^12 was 100% effective in preventing HW disease for 12 months in 236 dogs, of which 52 (22%) were recruited from the lower Mississippi River valley (LMRV), where ML resistance has been confirmed to occur [23]. In the same study, there were four dogs (4 of 218) in the positive control group, Heartgard^®^Plus, that developed heartworm infections, with all four cases coming from the LMRV, suggesting the reason for the preventive failures may have been due to exposure to ML-resistant HWs.

However, in spite of the encouraging activity of moxidectin in topical and injectable formulations, as with other MLs, the dosages tested to date for oral moxidectin have provided incomplete protection in dogs against ML-resistant *D. immitis* strains. The aim of the three studies reported here was to investigate whether increasing the oral moxidectin dose and increasing the number of consecutive monthly treatments would improve efficacy against ML-resistant *D. immitis* strains.

## Methods

### Study guidelines

Studies were conducted in accordance with the Center for Veterinary Medicine (CVM) Guidance for Industry #90, Effectiveness of Anthelmintics: General Recommendations (VICH guideline GL7) [40] and CVM Guidance for Industry #113, Efficacy of Anthelmintics-Specific Recommendations for Canines (VICH guideline GL19) [41].

### Study design

Three masked, negative, placebo-controlled, randomized studies were designed to evaluate the efficacy of different doses and dosage regimens of oral moxidectin in preventing HW disease against three ML-resistant *D. immitis* strains, namely JYD-34, ZoeMO and ZoeLA in dogs.

#### Study 1

A total of 48 dogs (24 female, 24 male) were inoculated with 50 *D. immitis* JYD-34 third-stage larvae (L3) on Day − 30. Dogs were allocated to treatment groups and pens following a randomized block design, with block based on Day − 8 body weight and pen location. Dogs were treated with either placebo [an empty hydroxypropyl methycellulose (HPMC) capsule] (T01) or HPMC capsules filled with the appropriate amount of hand-pulverized ProHeart^®^ tablets to deliver a dose of 3, 6, 12 or 24 µg/kg moxidectin on Day 0 only (T02–T05) or on Days 0, 30 and 60 (Groups T01 and T06) (Table 1). All dogs were necropsied for recovery and enumeration of adult HWs on Day 122 (152 days post-inoculation). The results generated as part of this study for the 3 µg/kg groups (T02 and T06) and the placebo group (T01) have been previously published [32] but are being reported again here for completeness and to provide background for the rationale of the study design.

**Table 1:** Study 1 design and efficacy of oral moxidectin against *Dirofilaria immitis* (JYD-34)

Group^a^ (*n* = 8)	Treatment^b,c^	Dosage (µg/kg)	Days of treatment	No. of dogs with worms	Adult *D. immitis* worm counts^d^		
					Individual worm counts	Geometric mean^e^	% reduction
T01	Placebo	na	0, 30, 60	8 of 8	29, 32, 33, 35, 36, 39, 43, 43	35.9^g^	–
T02^f^	Moxidectin	3	0	8 of 8	20, 25, 27, 29, 30, 32, 35, 39	29.1^h^	19.0
T03	Moxidectin	6	0	8 of 8	19, 21, 24, 25, 26, 28, 35, 43	26.8^h^	25.5
T04	Moxidectin	12	0	8 of 8	17, 20, 20, 23, 23, 26, 33, 35	24.0^h,i^	33.3
T05	Moxidectin	24	0	8 of 8	7, 13, 16, 16, 19, 20, 24, 29	16.8^i^	53.2
T06^f^	Moxidectin	3	0, 30, 60	8 of 8	7, 13, 18, 21, 25, 27, 29, 36	20.0^h,i^	44.4

^a^All dogs were inoculated with 50 *D. immitis* L3 (JYD-34 strain) at Day -30

^b^To maintain masking, dogs in T02, T03, T04 and T05 were administered an empty hydroxypropyl methycellulose (HPMC) capsule on Days 30 and 60

^c^Moxidectin administered as HPMC capsules filled with the appropriate amount of hand-pulverized ProHeart^®^ tablets to deliver the correct dose

^d^All dogs were necropsied for recovery and enumeration of adult heartworms on Day 122 (140 days post-inoculation)

^e^Means with different superscript letters (g–i) are significantly different (2.51 ≤ *t*_*df*_ ≤ 5.28, *P* ≤ 0.0342, where 4.65 ≤ *df* ≤ 8.61)

^f^Conducted as part of this study but first reported in McTier et al. [32]

*Abbreviation*: na, not applicable

#### Study 2

Fifty dogs (25 female, 25 male) were allocated to treatment following a randomized block design within room, with block based on pen location within room and Day − 35 body weight. Ten groups (*n* = 5) of dogs were inoculated with 50 *D. immitis* L3 on Day − 30; four groups (T01, T03–T05) were given the JYD-34 strain and six groups (T02, T06–T10) were given the ZoeLA strain. On Day − 3, dogs were moved to their allocated treatment pens. Treatment consisted of either a placebo (a tablet containing inert ingredients) or oral moxidectin (ProHeart^®^ tablets shaved to deliver the correct dose). Placebo tablets were administered on Days 0, 28 and 56 (T01, T02). Moxidectin was administered at 3 (T06), 24 (T03, T07), 40 (T04, T08) or 60 µg/kg (T05, T09, T10), and treatments were given either on Day 0 only (T06 and T09) or on Days 0, 28 and 56 (T03, T04, T05, T07, T08, T10) (Table 2). Efficacy of moxidectin in preventing HW infection was evaluated at Day 103 (133 days post-inoculation) following necropsy and adult worm recovery and enumeration.

**Table 2:** Study 2 design and efficacy of oral moxidectin against *Dirofilaria immitis* (JYD-34, ZoeLA)

Strain	Group^a^ (*n* = 5)	Treatment^b^	Dosage (µg/kg)	Days of treatment	No. of dogs with worms	Adult *D. immitis* worm counts^c^		
						Individual worm counts	Geometric mean^d^	% reduction^e^
JYD-34	T01	Placebo	na	0, 28, 56	5 of 5	5, 23, 27, 33, 33	20.6^f^	–
	T03	Moxidectin	24	0, 28, 56	1 of 5	2	0.2^g^	98.8
	T04	Moxidectin	40	0, 28, 56	0 of 5	0	0^g^	100.0
	T05	Moxidectin	60	0, 28, 56	0 of 5	0	0^g^	100.0
ZoeLA	T02	Placebo	na	0, 28, 56	5 of 5	25, 26, 31, 37, 38	30.9^f^	–
	T06	Moxidectin	3	0	5 of 5	11, 13, 17, 19, 25	20.0^g^	44.4
	T07	Moxidectin	24	0, 28, 56	1 of 5	1	0.1^h^	99.5
	T08	Moxidectin	40	0, 28, 56	1 of 5	1	0.1^h^	99.5
	T09	Moxidectin	60	0	5 of 5	1, 1, 6, 6, 10	3.6^i^	88.2
	T10	Moxidectin	60	0, 28, 56	0 of 5	0	0^h^	100

^a^All dogs were inoculated with 50 *D. immitis* L3 at Day -30. Groups T01 and T03–T05 received the JYD-34 strain; Groups T02 and T06–T10 received the ZoeLA strain

^b^Moxidectin administered as ProHeart^®^ tablets shaved to deliver the correct dose

^c^All dogs were necropsied for recovery and enumeration of adult heartworms on Day 103 (133 days post-inoculation)

^d^Means with different superscript letters (f–i) are significantly different within each strain *t*_(34)_ = 2.33, *P* = 0.0259 for T02 *vs* T06; 7.36 ≤ *t*_(34)_ ≤ 13.21, *P *< 0.0001 for all other significant differences

^e^Percentage reduction is compared to T01 for Groups T03–T05 and compared to T02 for Groups T06–T10

*Abbreviation*: na, not applicable

#### Study 3

Twenty-five dogs (15 female, 10 male) were allocated to five treatment groups (*n* = 5) and pens according to a randomized block design. Block was based on pen location and Day − 34 body weights. All animals were inoculated with 50 *D. immitis* L3 on Day − 28 (ZoeMO strain) and then treated with either a placebo (tablet containing inert ingredients) or oral moxidectin (ProHeart^®^ tablets shaved to deliver the correct dose). Placebo tablets were given on Days 0, 28 and 56. Moxidectin treatments were administered on Day 0 only at 3 µg/kg (T02) or on Days 0, 28 and 56 at 24 (T03), 40 (T04) or 60 µg/kg (T05) (Table 3). Preventive efficacy was evaluated at Day 120 (148 days post-inoculation) following necropsy and adult worm recovery and enumeration.

**Table 3:** Study 3 design and efficacy of oral moxidectin against *Dirofilaria immitis* (ZoeMO)

Group^a^ (*n* = 5)	Treatment^b^	Dosage (µg/kg)	Days of treatment	No. of dogs with worms	Adult *D. immitis* worm counts^c^		
					Individual worm counts	Geometric mean^d^	% reduction
T01	Placebo	N/A	0, 28, 56	5 of 5	23, 31, 31, 34, 36	30.7^e^	–
T02	Moxidectin	3	0	5 of 5	2, 3, 5, 9, 15	5.5^f^	82.1
T03	Moxidectin	24	0, 28, 56	1 of 5	1	0.1^g^	99.5
T04	Moxidectin	40	0, 28, 56	0 of 5	0	0^g^	100
T05	Moxidectin	60	0, 28, 56	0 of 5	0	0^g^	100

^a^All dogs were inoculated with 50 *D. immitis* L3 (ZoeMO strain) at Day − 28

^b^Moxidectin administered as ProHeart^®^ tablets shaved to deliver the correct dose

^c^All dogs were necropsied for recovery and enumeration of adult heartworms on Day 120 (148 days post-inoculation)

^d^Means with different superscript letters (e–g) are significantly different (7.34 ≤ *t*_(16)_ ≤ 16.0, *P* < 0.0001)

*Abbreviation*: na, not applicable

### Animals

Individually identified, purpose-bred intact male and female beagles 7–18 months of age at the time of infection were used in all three studies. All animals received a physical examination by a veterinarian to determine health and suitability for inclusion in the study. Animals weighed between 6.8–14.3 kg at the time of study initiation and were acclimated for 5–9 days prior to inoculation.

All dogs were housed individually within a mosquito-proof facility in indoor cages that complied with accepted animal welfare legislation and guidance. They were fed an appropriate maintenance diet of a commercial dry canine ration and had access to water *ad libitum*. Standard environmental conditions were maintained and environmental enrichment and social interactions were provided. Treatment groups (*n* = 8, Study 1; *n* = 5, Studies 2 and 3) consisted of approximately equal numbers of male and female dogs. No dog had ever received ProHeart^®^ 6 or any ML-containing product within 90 days prior to the start of the study.

Dogs were determined to be free of HW infection by modified Knott’s test, and commercially available adult HW antigen tests (DiroCHEK^®^ or WITNESS^®^ Heartworm Antigen Test Kit, Zoetis, Parsippany; SNAP Heartworm RT Test, Idexx, Westbrook, ME, USA; Solo Step^®^ Canine Heartworm Test, Heska, Loveland, CO, USA) at least 1 week prior to L3 inoculation. Heat treatment of serum prior to HW antigen testing was not performed. To identify any infections not previously detectable at study start, additional blood samples were collected on Day 59 (Studies 1 and 3) or Day 60 (Study 2) and examined for *D. immitis* microfilariae and tested for adult *D. immitis* antigen. In Study 1, blood samples were also collected on Days 104 and 122 and examined for adult *D. immitis* antigen to detect infection due to experimental inoculation.

### Heartworm strains

The HW strains used in the studies were derived from isolates recently (within the previous 5 years of initial testing) collected from naturally infected dogs from the middle to southeastern USA. JYD-34 (Studies 1 and 2) was originally collected from a naturally occurring field case of canine HW in Illinois in July 2010. ZoeMO-2012 (ZoeMO) (Study 3) was collected in December 2012 from the same dog from which the original JYD-34 strain had been collected 2.5 years earlier (J. McCall, personal oral communication, 2013). The dog had been maintained in mosquito-proof quarters with no additional administration of macrocyclic lactones during the intervening time. ZoeLA (Study 2) was collected from a naturally occurring field case of canine HW in Louisiana in June 2013. All three isolates were validated as infective strains through the diagnosis of circulating microfilariae, positive heartworm antigen test results and adult worm recovery in recipient animals. Additional details on these strains are provided by McTier et al. [32].

### *Dirofilaria immitis* inoculations

Dogs in all studies were administered 50 viable L3 in RPMI balanced media solution by subcutaneous injection in the inguinal region. Larvae were harvested from infected *Aedes aegypti* mosquitoes reared and maintained at Zoetis (Kalamazoo, MI, USA) as previously described [6]. Studies were conducted within 4 years of the heartworm isolate collection from the field.

### Treatments

For all studies, moxidectin was supplied as commercial ProHeart^®^ tablets (30, 68 and 136 µg of moxidectin/tablet) that were obtained as commercial products directly from the Zoetis manufacturer (Fort Dodge, IA, USA); these tablets were produced for markets in Japan and/or Australia. The correct size and number of tablets were selected based on the total dosage of moxidectin needed. Tablets were pulverized and placed in HPMC capsules or shaved to deliver the exact calculated point dose of moxidectin, based on individual body weights obtained within 8 days of each treatment. In all studies, capsules/tablets were administered by mouth along with 5 ml of water to encourage swallowing. Treatment was carried out following overnight fasting, and each dog was then offered its regular food ration within 2 h of dosing. Dogs were observed for several minutes after dosing to confirm that the dose was swallowed and to monitor for potential adverse events.

#### Study 1

Dogs in the moxidectin-treated groups (Groups T02–T06) were administered capsules filled with the appropriate amount of hand-pulverized moxidectin, with dosage calculated based on the individual body weights recorded on Day − 8 (Day 0 dosing), Day 23 (Day 30 dosing) or Day 53 (Day 60 dosing). The moxidectin-filled capsules were administered to dogs in T02–T05 on Day 0 only and to dogs in T06 on Days 0, 30 and 60 (see Table 1). To maintain masking, dogs in T02–T05 were administered an empty capsule on Days 30 and 60, and dogs in the control group (T01) were administered an empty capsule on Days 0, 30 and 60.

#### Study 2

Dogs in the moxidectin-treated groups (T03–T10) were administered ProHeart^®^ tablets shaved/sanded to the appropriate dose for the body weight determined for each individual dog on Day − 3 (Day 0 dosing), Day 25 (Day 28 dosing) or Day 53 (Day 56 dosing). The moxidectin tablets were administered to dogs in T06 and T09 on Day 0 only and to dogs in T03–T05, T07–T08 and T10 on Days 0, 28 and 56 (see Table 2). Dogs in T01 and T02 were administered one whole placebo tablet (containing vehicle only) on Days 0, 28 and 56.

#### Study 3

Dogs in the moxidectin-treated groups (T02–T05) were administered ProHeart^®^ tablets sanded/shaved so that each dog received the appropriate dose based on the individual body weight recorded on Day − 5 (Day 0 dosing), Day 27 (Day 28 dosing) or Day 55 (Day 56 dosing). Dogs in T03–T05 were dosed on Days 0, 28 and 56, whereas dogs in T02 were dosed on Day 0 only (Table 3). Dogs in T01 were administered one whole placebo tablet on Days 0, 28 and 56.

### Animal observations

Physical examinations were performed by a veterinarian immediately prior to the start of each study (Day − 60, − 35 or − 34 for Study 1, 2 or 3, respectively) and included, but were not limited to, rectal temperature, thoracic auscultation, skin and hair coat assessment, and the general physical condition of each dog. General health observations were made for each dog either once (Studies 2 and 3) or twice (Study 1) daily. Clinical observations were made on all animals prior to and at 1, 3, 6 and 24 h after the administration of vehicle or test product on Days 0, 30 and 60 (Study 1) or on Days 0, 28 and 56 (Studies 2 and 3). All personnel conducting observations were masked to treatment allocations and changed protective clothing between handling each dog.

### Necropsy and parasite recovery

Dogs were humanely euthanized after an intravenous injection of heparin *via* an approved pentobarbital euthanasia solution on Day 122 (Study 1), Day 103 (Study 2) or Day 120 (Study 3). The pleural and peritoneal cavities were examined for adult *D. immitis*, the posterior and anterior vena cavae were clamped, and the heart and lungs were removed. The precava, right atrium, right ventricle and pulmonary arteries (including those coursing through the lungs) were dissected and examined. Any adult worms present were recovered and classified as male or female and as either dead (worms abnormal in both appearance and motility) or alive (all other worms) as previously described [42]. Dogs were randomly assigned to order of euthanasia and necropsy.

### Data analysis

The experimental unit for treatment was the individual dog. Within each study, the numbers of adult *D. immitis* recovered during post mortem examinations were summarized for each treatment group. The natural [log_e_ (x + 1)] transformation was applied to all counts prior to analysis, and the geometric means (back-transformed least squares means) were calculated.

Percent efficacy, relative to the control group and based on geometric means, was calculated as follows:

$$ {\text{\%  Efficacy}} = \frac{{\left( {{\text{Mean Control}} - {\text{Mean Treated}}} \right)}}{\text{Mean Control}} \times 100 $$

Treatment differences were assessed between control and treated groups using contrasts in a general linear mixed model analysis of natural logarithm transformed worm counts and a significance level of α = 0.05. The models included the fixed effect of treatment and random effects of room (study 2 only), block within room, and error. SAS PROC MIXED was used to fit the statistical models and the Kenward–Roger option was used to calculate degrees of freedom for the test-statistics used for pairwise treatment comparisons. All analyses were carried out using SAS/STAT Release 9.3 (SAS Institute, Cary, NC, USA).

## Results

### Efficacy

In each study, control dogs inoculated with each *D. immitis* strain were positive for adult worms at necropsy, and worm counts confirmed the adequacy of the infections (Tables 1, 2, 3). Geometric mean (range) HW counts for untreated dogs inoculated with the JYD-34 strain were 35.9 (29–43) in Study 1 (T01) and 20.6 (5–33) in Study 2 (T01). Control dogs inoculated with ZoeLA in Study 2 (T02) and ZoeMO in Study 3 (T01) harbored a geometric mean (range) of 30.9 (25–38) and 30.7 (23–36) adult HWs, respectively.

In all three studies, all dogs administered moxidectin (at every dose and with every regimen) showed significantly reduced adult worm counts compared to control dogs (Study 1, 2.51 ≤ *t*_*df*_ ≤ 5.28, *P* ≤ 0.0342, where 4.65 ≤ *df* ≤ 8.61; Study 2, 7.36 ≤ *t*_(34)_ ≤ 13.21, *P* < 0.0001 for all treatments except for a single dose of moxidectin at 3 µg/kg (T02), *t*_(34)_ = 2.33, *P* = 0.0259; Study 3, 7.34 ≤ *t*_(16)_ ≤ 16.0, *P* < 0.0001) (Tables 1, 2, 3).

In Study 1, dogs treated with a single dose of moxidectin at 3 µg/kg 30 days after inoculation with *D. immitis* JYD-34 L3 showed a 19.0% reduction in mean worm counts compared with control dogs. Administering the same dose for three consecutive months increased the level of protection to 44.4%, higher than that achieved with a single dose of moxidectin at 12 µg/kg (33.3%) and approaching the efficacy of one dose at 24 µg/kg (53.2%) (Table 1); however, all dogs in all groups were infected with HW regardless of dose and regimen used. In Study 2, efficacy of moxidectin against JYD-34 was further improved when dogs were given moxidectin for three consecutive months; > 98% efficacy was obtained at 24 µg/kg (T03) with only 1 of 5 dogs infected, and 100% efficacy was demonstrated with three treatments of both 40 (T04) and 60 µg/kg (T05) (Table 2).

Dogs inoculated with the *D. immitis* ZoeMO strain (Study 3) showed similar results to those obtained using the JYD-34 strain when treated with three consecutive monthly dosages of moxidectin; only one dog treated with 24 µg/kg moxidectin had a single worm at necropsy, and a dose of either 40 (T04) or 60 µg/kg (T05) was 100% effective in preventing the development of *D. immitis* (Table 3).

Treating with three dosages of moxidectin at 24 or 40 µg/kg (Study 2, T07 and T08, respectively) provided > 99% efficacy in dogs inoculated with the ZoeLA strain, with 1 of 5 dogs infected. A similar pattern was observed at 60 µg/kg, with a single dose (T09) providing 88.2% efficacy (5 of 5 dogs infected) and three dosages (T10, Study 2) providing complete prevention in all dogs.

### Health observations

There were no mortalities among the dogs involved in any of the three studies. Various abnormal health observations were made sporadically during these studies across all treatment groups (e.g. emesis, soft stool, diarrhea, abrasions, broken toenail, interdigital cyst, prolapsed nictitans gland). Most signs resolved with either no or minimal treatment (e.g. temporary anti-inflammatory or antimicrobial administration). All clinical signs recorded during the studies were deemed to be unrelated to treatment with moxidectin.

## Discussion

The increasing number of HW LOE reports in dog populations across the USA is well documented. Between 2000 and 2002, the number of LOE cases reported to the US Food and Drug Administration, Center for Veterinary Medicine rose from 405 to 951, and the following 12 months (2002–2003) saw another increase to 1503 [22]. Twenty years after the first LOE reports, analyses to determine patterns of LOE are being conducted on more than 45,000 LOE cases reported between 2004 and 2015 with preliminary results available [24]. The exact role of ML resistance in these LOE cases remains unclear, but with more than 15 ML-resistant strains already identified and all presently marketed MLs showing less than 100% efficacy against at least one of these strains in laboratory studies, it is apparent that resistance in *D. immitis* strains to MLs is real and has the potential to become more widespread [26, 27, 30, 32, 33, 43]. It is therefore important that new treatment options be available to protect dogs exposed to *D. immitis* populations with reduced susceptibility to MLs.

The three strains used in the present evaluations, JYD-34, ZoeMO and ZoeLA, have been well characterized as ML-resistant [32, 36, 37]. Additionally, data reported here and in previous publications show differences in the strains’ susceptibilities to moxidectin and other MLs [25, 27, 32, Tom L. McTier, pers.comm.]. As reported by McTier et al. [32], the efficacy of a single oral dose of moxidectin at 3 µg/kg varied greatly across the strains, with efficacies of 19.0% for JYD-34, 44.4% for ZoeLA and 82.1% for ZoeMO, while six consecutive monthly oral dosages of milbemycin oxime (0.5 mg/kg) was only 72.2% effective against the JYD-34 resistant strain ([44]; Table 4) and three monthly dosages of oral ivermectin (6 µg/kg) was only 29.0% effective against this same strain [25] (Table 4). The variations in susceptibility to moxidectin and other MLs can likely be explained by genetic differences displayed among the strains. The identification of 42 single nucleotide polymorphisms (SNPs) with alternative alleles known to be associated with ML resistance has shed some light on this phenomenon, with frequency of specific alternative alleles being associated with the strength of the resistant phenotype [27, 36]. Using a pool of 16 *D. immitis* strains (including JYD-34, ZoeMO and ZoeLA) well characterized in terms of their response to MLs, Bourguinat et al. [36] produced a heat map of ML response and the frequency of the alternative allele at all of the 42 SNP positions and identified nine SNPs most strongly correlated with ML phenotypic response. For the three strains used in our studies, the heat map shows all (JYD-34) or almost all (ZoeMO, ZoeLA) of these 9 SNPs with increased frequencies of the alternative alleles, although across the three strains there are differences in the frequency of the alternative alleles represented at each SNP. Particularly interesting are the efficacy and genetic differences observed between JYD-34 and ZoeMO, which were isolated from the same dog with ZoeMO isolated 2.5 years after the original JYD-34 strain was taken, suggesting potential reversion of resistance to a more susceptible state over time [32, 36, 37, 45]. These differences warrant further study to understand efficacy variations among resistant strains and how resistance might fluctuate in a dynamic population under drug pressure.

**Table 4:** Summary of macrocyclic lactone preventives for efficacy against *Dirofilaria immitis* (JYD-34 strain) in dogs

Compound	Day inoculated with L3^a^	Treatment regimen		Results			Reference
		Macrocyclic lactone dose	Day	No. of dogs with worms	Mean^b^ (range)	Efficacy (%)	
Moxidectin (ProHeart^®^ tablets)	− 30	3 µg/kg	0	8 of 8	29.1 (20–39)	19.0	McTier et al. [32]; this paper
	− 30	3 µg/kg	0, 30, 60	8 of 8	20.0 (7–36)	44.4	McTier et al. [32]; this paper
	− 30	6 µg/kg	0	8 of 8	26.8 (19–43)	25.5	This paper
	− 30	12 µg/kg	0	8 of 8	24.0 (17–35)	33.3	This paper
	− 30	24 µg/kg	0	8 of 8	16.8 (7–29)	53.2	This paper
	− 30	24 µg/kg	0, 28, 56	1 of 5	0.2 (0–2)	98.8	This paper
	− 30	40 µg/kg	0, 28, 56	0 of 5	0	100	This paper
	− 30	60 µg/kg	0, 28, 56	0 of 5	0	100	This paper
Moxidectin—injectable (ProHeart^®^ 6)	2	0.17 mg/kg	0	1 of 6	0.1 (0–1)	99.5	Bowman et al. [29]
Moxidectin—topical (Advantage^®^ Multi: imidacloprid/moxidectin)	− 30	2.8–6.7 mg/kg	0	0 of 8	0	100	Blagburn et al. [25]^c^
Ivermectin—oral (Heartgard^®^Plus Chewables for Dogs: ivermectin/pyrantel pamoate)	− 30	6.2–11.9 μg/kg	0, 31, 60	8 of 8	13.1 (5–19)	29.0	Blagburn et al. [25]
Selamectin—topical (Revolution^®^)	− 30	6.6–13.1 mg/kg	0, 31, 60	8 of 8	8.8 (3–18)	28.8	Blagburn et al. [25]
Milbemycin oxime—oral (Trifexis^®^ Chewables for Dogs: milbemycin oxime/spinosad)	− 30	0.5–1.0 mg/kg	0, 31, 60	8 of 8	13.1 (9–18)	52.2	Blagburn et al. [25]
Milbemycin oxime—oral (Interceptor^®^: milbemycin oxime)	− 30, − 23^d^	0.92 ± 0.15 mg/kg^e^	0	8 of 8	9.3 (0–39)	58.2	McCall et al. [55]
Milbemycin oxime (NexGard Spectra^®^)	− 30	0.5–1.0 mg/kg	0, 30	na	na	76.1	NexGard Spectra^®^ EPAR, 2014 [44]
Milbemycin oxime (NexGard Spectra^®^)	− 30	0.5–1.0 mg/kg	0, 30, 60	na	na	72.2	NexGard Spectra^®^ EPAR, 2014 [44]
Milbemycin oxime (NexGard Spectra^®^)	− 30	0.5–1.0 mg/kg	0, 30, 60, 90, 120, 150	na	na	72.0	NexGard Spectra^®^ EPAR, 2014 [44]

^a^Day of first treatment is designated as Day 0. All dogs inoculated with 50 L3 unless otherwise specified

^b^Geometric means (arithmetic means for McCall et al. [55])

^c^Less than 100% efficacy was obtained with 2.5 mg/kg topical moxidectin in a similar study (Dr John McCall, personal communication)

^d^Dogs infected with an estimated total of 78 ± 14 L3 *via* two exposures to infected mosquitoes

^e^Mean dose administered

*Abbreviations*: EPAR, European Public Assessment Report; na, data not available

Historically, moxidectin has been shown to be the most potent of the MLs in preventing the development of *D. immitis* in dogs when compared across various effectiveness studies [8, 46–48]. As originally tested, a single oral dose of only 0.5 µg/kg moxidectin provided 100% prevention of HW development when administered 60 days after larval inoculation [8]. The higher preventive efficacy against *D. immitis* provided by moxidectin likely rests in its inherent potency and its physicochemical and pharmacokinetic properties [39, 49]. Moxidectin generally has a longer elimination half-life and larger volume of distribution than both ivermectin and milbemycin [50–52]. These characteristics result in moxidectin being distributed into host tissues, especially adipose tissue [53]. Moxidectin in adipose tissue remains active against migrating stages of parasites such as HW larvae. Over time, the moxidectin that was distributed to tissue will return to systemic circulation as active compound prior to elimination [2, 3, 39, 54]. Data in cattle suggest there is a high correlation between the plasma and adipose tissue concentration of moxidectin [53]. This results in longer persistence in the target host and helps explain the increased duration of efficacy compared to other MLs [49]. Collectively these attributes may explain the greater activity of moxidectin against HW larvae in the face of emerging ML resistance compared to other MLs.

The present studies demonstrated that increasing both the dose and the frequency (consecutive monthly dosages) of oral moxidectin increased the efficacy against all three ML-resistant HW strains (JYD-34, ZoeLA and ZoeMO) evaluated, with three consecutive monthly dosages of ≥ 40 µg/kg moxidectin providing complete preventive efficacy. Additionally, three dosages of moxidectin at 24 µg/kg was highly effective (≥ 98.8%) in preventing the development of all three HW strains, with only a single dog in each group (1 of 5) having just one or two worms. This high level of protection against ML-resistant *D. immitis* strains provided by repeated dosages has not been reported for any of the present commercially available oral preventives (only 29.0% and ≤ 72.2% efficacy with three consecutive monthly dosages of ivermectin or three to six consecutive monthly dosages of milbemycin oxime respectively; Table 4).

Furthermore, based on the existing very high efficacy (≥ 98.8%; 4 of 5 dogs heartworm free) obtained after three months of dosing with 24 µg/kg moxidectin against all three resistant strains and the unique attributes of the moxidectin molecule, it is likely that four or more consecutive monthly dosages of 24 µg/kg moxidectin may provide additional protection. This dose should provide robust HW preventive efficacy against the strains to which most dogs in the USA will be exposed, including *D. immitis* populations with reduced susceptibility to MLs, with an adequate margin of safety in the most sensitive patient population (collies). For these reasons, 24 µg/kg moxidectin was selected as the optimal dose for further commercial development in a new oral HW preventive.

## Conclusions

Increasing both the dose and the number of consecutive monthly oral dosages of moxidectin resulted in increased preventive efficacy against ML-resistant *D. immitis* with complete protection obtained using three dosages of moxidectin at either 40 µg/kg (JYD-34, ZoeMO) or 60 µg/kg (ZoeLA). Three consecutive monthly dosages of 24 µg/kg moxidectin provided very high efficacy (≥ 98.8% efficacy with 4 out of 5 dogs heartworm-free) against all three ML-resistant strains. Moxidectin administered at 24 µg/kg was selected as the optimal oral dose to develop as a safe and effective means of protecting dogs against HW disease in the face of increasing ML resistance.

## Data Availability

All relevant data supporting the conclusions of this article are included within the article.

## Abbreviations

ML: macrocyclic lactone
HW: heartworm
AHS: American Heartworm Society
LOE: lack of efficacy
JYD-34: *Dirofilaria immitis* strain JYD-34
ZoeLA: *Dirofilaria immitis* strain ZoeLA-2013
AMAL: *Dirofilaria immitis* strain AMAL-2014
ZoeMO: *Dirofilaria immitis* strain ZoeMO-2012
LMRV: lower Mississippi River valley
CVM: Center for Veterinary Medicine
L3: third-stage larvae
HPMC: hydroxypropyl methycellulose
SNP: single nucleotide polymorphism
